# The ERK-1 function is required for HSV-1-mediated G1/S progression in HEP-2 cells and contributes to virus growth

**DOI:** 10.1038/s41598-017-09529-y

**Published:** 2017-08-23

**Authors:** Ivana Colao, Rosamaria Pennisi, Assunta Venuti, Michaela Nygårdas, Outi Heikkilä, Veijo Hukkanen, Maria Teresa Sciortino

**Affiliations:** 1 Department of Biological and Environmental Sciences, University of Messina, Viale F. Stagno d’Alcontres 31, 98166 Messina, Italy; 2 Department of Virology, University of Turku, Turku, Finland; 3 Division of Immunology, Transplantation and Infectious Diseases, San Raffaele Scientific Institute, Milan, Italy

## Abstract

The herpes simplex virus 1 is able to readdress different cellular pathways including cell cycle to facilitate its replication and spread. During infection, the progression of the cell cycle from G1 to S phase makes the cellular replication machinery accessible to viral DNA replication. In this work we established that HSV-1, in asynchronized HEp-2 cells, strictly controls cell cycle progression increasing S-phase population from 9 hours post infection until the end of HSV-1 replication. The G1/S phases progression depends on two important proteins, cyclin E and CDK2. We demonstrate that their phosphorylated status and then their activity during the infection is strongly correlated to viral replication events. In addition, HSV-1 is able to recruit and distribute ERK1/2 proteins in a spatio-temporal fashion, highlighting its downstream regulatory effects on cellular processes. According with this data, using chemical inhibitor U0126 and ERK dominant negative cells we found that the lack of ERK1 activity affects cyclin E protein accumulation, viral gene transcription and percentage of the cells in S phase, during the viral replication. These data suggested a complex interaction between ERK, cell cycle progression and HSV-1 replication.

## Introduction

The herpes simplex virus type 1 (HSV-1) is a double stranded DNA virus belonging to the Herpesviridae family, known to be an excellent model to learn how the complex relations between the virus and the host cell are regulated. Indeed, during productive infection, HSV-1 dramatically remodels the architecture and physiology of the host cell, by interfering with the host-signaling machinery^1–4^. Early studies have shown that cellular factors expressed during G1/S phase efficiently support viral replication^5^. Others have demonstrated that immediate-early genes (IE) are specifically activated when cells are released from a serum starvation-induced growth arrest^6^. In addition, it has been demonstrated that the use of specific inhibitors of CDKs involved in the G1/S phase progression, results in substantial inhibition of Immediate Early (IE) and Early (E) HSV genes^2, 7, 8^. Thus, the activation of CDKs, potentially involved in the transition from G1 to S phases, seems to be necessary for the transcription and replication of viral DNA of HSV-1^2, 4, 5^. The involvement of IE regulatory proteins such as ICP0, ICP27, ICP4 and ICP22 is also required in the modification of cell cycle regulation in HSV infected cells^9–11^. In particular, other authors have demonstrated the association of CDK and cyclin proteins with the herpes simplex virus infection. These studies demonstrated the important role that ICP0 plays during cell cycle regulation. ICP0 monitors the function of cyclin type D and is able to stabilize the cyclin D3^12–14^, modulating the cyclin D3 levels in a critical homeostatic level^15^. It has been shown that a single amino acid mutation in ICP0 abolishes the ability of ICP0 to interact with cyclin D3, compromising the ability of a corresponding mutant virus to replicate in serum-deprived/arrested cells, but not in proliferating cells^15, 16^.

Accumulating evidence suggests that cell cycle progression, strictly correlated to CyclinE/CDK2 activity, is dependent on the MEK-ERK kinase cascade. The initial evidence linking ERK1/2 signaling to cell growth control stemmed from the finding that PD98059 inhibitor blocks the stimulation of global cellular protein synthesis. Subsequent data have shown that the nuclear-localized CDK2, co-expressed with cyclin E, requires ERK activity, following mitogenic stimulation, as a second role for ERK in G1 progression^17–19^. It is well known that viruses manipulate host MAPK signaling pathways to stimulate their productive replication, control cell proliferation or suppress programmed cell death^20–23^.

Herpes simplex virus type 1 (HSV-1), which induces profound changes in cellular pathways in infected cells, depending on the cellular model, is able to regulate the MAPK pathways positively or negatively^24–30^.

To further define the cellular environment and considering the importance of ERK in regulating CDK2 phosphorylation^31^ we examined the effects of HSV-1 replication on cell cycle distribution and the activity of cyclin E/CDK2 complex in HEp-2 permissive cell line. We investigated the recruitment of ERK signaling as a key factor in controlling cell cycle progression mediated by HSV-1 and its impact on viral replication. We report here significant differences in the percentage of cells in the S phase of HEp-2 infected cells compared to the control. Consistent with this observation we saw that the increase in the S phase of HEp-2 infected cells correlates with the increased level of cyclin E phosphorylation. Finally, no increase in activity of cyclin E was observed in cells where the ERK pathway was inhibited either chemically or with a dominant negative ERK1 mutant. The results suggest that HSV-1 specifically maintains high levels of ERK activity, most likely to control cell cycle progression through the cyclin E/CDK2 complex, for its own advantage.

## Results

### Distribution of the S phase of cell cycle mediated by HSV-1 infection

**Figure 1:**
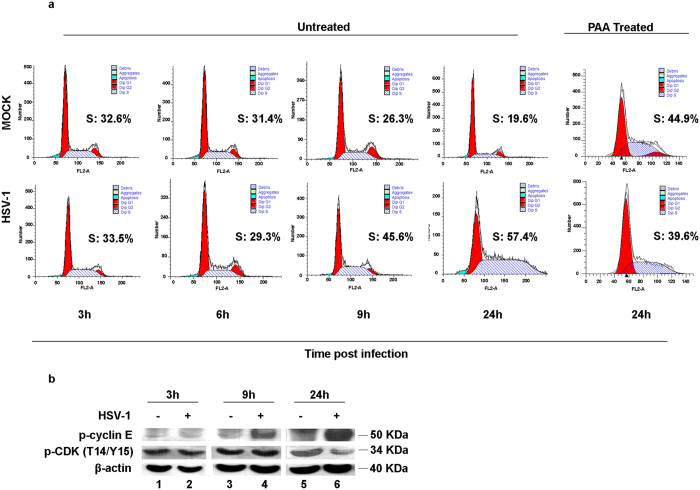
Cell cycle analysis and expression of p-﻿﻿cyclin E and p-CDK in HEp-2 cells during HSV-1 infection. (**a**) HEp-2 cells were mock infected or infected with HSV-1 at MOI 10, and collected at 3, 6, 9 and 24 h p.i. At 24 h cells were infected or mock infected incubated in presence of DNA polymerase inhibitor phosphonoacetic acid (PAA). The cellular DNA content was determined at indicated times by FACS analysis as described in Methods. Processed cells were labelled with PI (10 µg/ml) to calculate different phases of cell cycle. The data were analyzed as means of triplicate ± SD and the percentage of S-phase cells was obtained using ModFit LT 3.0 software. (**b**) HEp-2 cells were mock infected or infected with HSV-1 at MOI 10 and collected at 3, 9 and 24 h p.i. for proteins extraction. Equal amount of proteins were separated by polyacrylamide gel electrophoresis and probed with phospho-cyclin E (Thr 395)-R and phospho-CDK (T14/Y15) antibodies. β-actin was used as a loading control.

Studies of HSV-1 infected asynchronous cells have shown that at very early times post infection (p.i.) an “S-phase-like” environment is created^11^. However, the cellular pattern manipulated by the virus in this particular process is still unidentified. To solve this issue we examined the effects of HSV-1 replication on the progression of the cell cycle in asynchronously growing HEp-2 cells, fully permissive to viral replication. HEp-2 cells were exposed to 10 PFU of wild type HSV-1 and collected at different times p.i. (3, 6, 9 and 24 h). Samples were then stained with the fluorescent dye propidium iodide (PI), which is able to bind DNA with high affinity. The cells treated with the dye were analyzed using the Becton Dickinson FACScalibur flow cytometer and the results analyzed using the ModFit LT 3.0 software for the analysis of the cell cycle phase distribution, expressed in percentages of cells in G1, S, and G2/M phases. The results shown in Fig. 1a demonstrate striking differences in the percentage of cells in the S phase of infected cells compared to the control. In particular at 9 h p.i. it was possible to observe a higher percentage of infected cells in the S phase (45.6%) compared to uninfected (26.3%), underlining that when the viral genome is accumulating, the percentage of S-phase population increases compared to basal levels (*P* < 0,001). This effect was more marked at 24 h p.i., with 57.4% of infected cells in the S phase compared to uninfected cells (19.6%, *P* < 0,001). To establish that HSV infection is pushing cells into S phase, PAA treatment was used to block the accumulation of viral DNA. Figure 1a right panel, demonstrated a significant decrease (*P < *0.05) of the percentages in S phases in PAA treated and infected cells (39.6%) if compared to untreated HSV-1 infected cells (44.9%).

To explore the cellular molecules implicated in the regulation of the S phase transition recruited by HSV-1, we checked if the key regulators of cell cycle progression through the G1/S phase, cyclin E and cyclin dependent kinase 2 (CDK2) were involved. HEp-2 cells were infected with HSV-1 at MOI 10 and collected at different times after the infection (3, 9 and 24 h). Protein extracts of infected and uninfected cells were solubilized, transferred to a nitrocellulose sheet and probed with antibodies specific to phospho-cyclin E and phospho-CDK (T14/Y15). The results showed that the patterns of accumulation of cellular proteins in HSV-1-infected cells were different compared to the mock infected. In particular, we observed that the phosphorylated form of cyclin E is increased in HEp-2 cells infected with HSV-1, beginning at 9 h p.i. (Fig. 1b lane 4) and further increased by 24 hours, as compared to uninfected cells (Fig. 1b lane 6). Furthermore, at 9 and 24 h HSV-1 infection in HEp-2 cells leads to a decrease of the phosphorylated form of CDK protein. Data from literature demonstrated that the association with cyclin E and subsequent nuclear translocation of CDK2 requires the removal of two inhibitory phosphates (T14 and Y15)^32–34^. The loss of these inhibitory phosphate coincides with the activation of CDK2 and then with the assembly of the complex cyclin E/CDK2. Based on that it was possible to consider that, during the HSV-1 replication, high levels of cyclin E linked to reduction of CDK’s inactivated form are correlated to G1/S phase progression driven by HSV-1.

### Activation and nuclear translocation of ERK in response to HSV-1 infection

**Figure 2:**
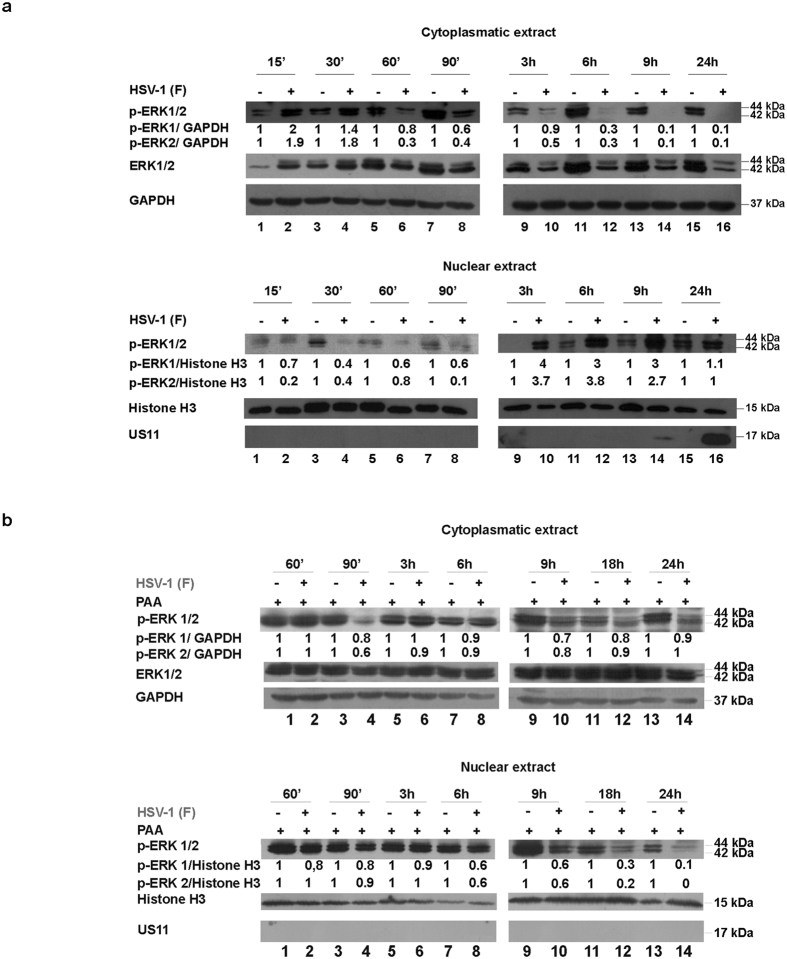
Expression of phospho-ERK1/2 proteins in HEp-2 cells infected with HSV-1 and PAA treated. (**a**) HEp-2 cells were infected or mock infected with HSV-1 at MOI 10 and collected at different times p.i. (15′, 30′, 60′, 90′, 3, 6, 9 and 24 hrs). Equal amount of cytoplasmic and nuclear proteins were separated by polyacrylamide gel electrophoresis and probed with phospho-ERK1/2 (p42/44), ERK1/2 and US11 antibody. GAPDH and Histone 3 proteins were used as housekeeping for the cytoplasmic and nuclear fraction, respectively. (**b**) Cells were infected or mock infected and treated with 300 µg/ml phosphonoacetic acid (PAA) and collected at 60′, 90′, 3, 6, 9 18 and 24 hrs. PAA was added at the time of adsorption and maintained throughout the course of infection and processed as described in panel b. Band density was determined with the T.I.N.A. program, and was expressed as fold change over the appropriate housekeeping genes.

Having identified the main key regulator of cell cycle progression during HSV-1 replication, the activated cyclin E/CDK2 complex, we next examined the recruitment of ERK signaling during HSV-1 replication in HEp-2 cells. To this end, a western blotting analysis of cytoplasmic and nuclear proteins was performed. HEp-2 cells were mock infected or infected with HSV-1 at MOI 10, 15′, 30′, 60′, 90′, 3, 6, 9 and 24 h p.i. Samples were subjected to cytoplasmic and nuclear proteins extraction for western blot analysis (as described in Methods). The results demonstrate that upon HSV-1 infection, an increase in phosphorylated ERK1/2 at the early phase of infection (15′) occurred, whereas it was reduced at the late phase of infection (24 h) (Fig. 2). To note, accumulation of the phosphorylated-ERK1/2 (p-ERK1/2) proteins were mainly localized into the cytoplasm during the early phase of HSV replication (Fig. 2a, lanes 2, 4, 6 and 8). In contrast, in the nuclear fractions of infected cells we noted an absence of the p-ERK1/2 proteins during the early phase of HSV infection (Fig. 2a, lanes 2, 4, 6 and 8). Unexpectedly, we observed a constant accumulation of p-ERK1/2 later, from 6 h through 24 h, matching with the active viral replication (Fig. 2a lanes 10, 12, 14 and 16). These data demonstrated that the enrollment of the ERK pathway is HSV-mediated in a spatio-temporal fashion and that the delay of translocation into the nucleus of phosphorylated ERK1/2 proteins established the capability of the virus to restrict activated ERK1/2 to the cytoplasm. To verify the contribution of active HSV-1-replication in the p-ERK1/2 accumulation and nuclear compartmentation, we infected or mock-infected HEp-2 cells with HSV-1 at MOI 10, in presence of DNA polymerase inhibitor phosphonoacetic acid (PAA, 300 µg/ml). PAA was added in presence of viral inoculum and cells were collected at 60′, 90′ 3, 6, 9, 18 and 24 h p.i. Equal amount of cytoplasmic and nuclear proteins were used for western blot analysis (as described in Methods) and probed with an antibody which recognize a phosphorylated form of ERK1/2 proteins (p42/44). The cytoplasmic protein GAPDH and the nuclear protein Histone H3 were used as housekeeping genes. The expression of US11, a γ_2_ gene, was used as a control of PAA treatment. Indeed, PAA is able to block the γ_2_ genes expression, during HSV-1 replication in cell cultures^35^. We report the unexpected finding that PAA behaves as strong inducer of ERK1/2 phosphorylation in both infected and uninfected HEp-2 wt cells with predominantly accumulation in cytoplasmatic fraction at all time considered. To note, although PAA induces ERK1/2 phosphorylation, in treated infected cells, p-ERK1/2 fails to accumulate in the nuclear compartment at all time considered (Fig. 2b, nuclear fraction, lanes 8,10,12 and 14). These data highlight that the inhibition of HSV-1 replication by PAA, block in turn the nuclear accumulation of pERK1/2 at late time, if compared to untreated infected cells shown in nuclear fraction of Fig. 2a, lanes 10,12,14 and 16.

### Effect of U0126 inhibitor on cyclin E and CDK2 accumulation

**Figure 3:**
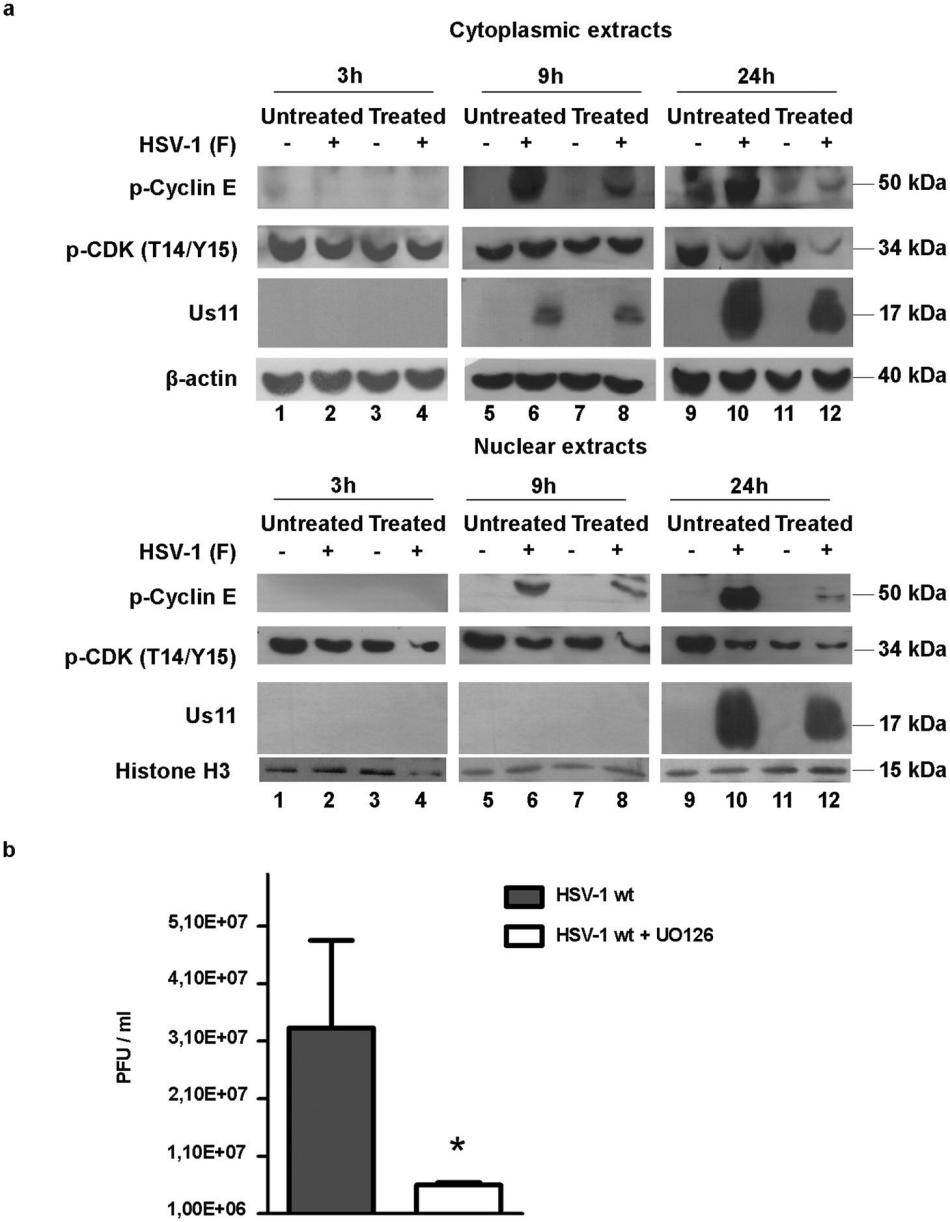
Effect of U0126 inhibitor treatment on cyclin E and CDK2 expression and on viral replication. HEp-2 cells treated or untreated cells with U0126 (5 µM) were mock-infected or infected with HSV-1 at MOI 10 and collected at 3, 9 and 24 h p.i. (**a**) The cytoplasmic and nuclear proteins extraction were obtained as described in Methods. Equal amount of cytoplasmic and nuclear proteins were separated by polyacrylamide gel electrophoresis and probed with phospho-cyclin E, phospho-CDK (T14-Y15) and Us11 antibodies. The β actin and Histone H3 were used as a loading control. (**b**) Virus yield was determined at 24 h pi by using the standard plaque assay on VERO cells. The data are shown as means of triplicate ± SD and *indicate significant changes (p value ≤0.001).

We next exploited the possible involvement of the extracellular signal-regulated kinases as the main regulator of cyclin E/CDK2 complex during HSV-1 replication. To this purpose, we used the U0126 inhibitor as a highly selective inhibitor of MAPKase. HEp-2 cells were pre-treated for 1 h with U0126 (5 μM) and then exposed to wild-type HSV-1 at MOI 10 in the presence of the inhibitor. At 1 h after exposure, the inoculum was replaced with fresh medium containing U0126. The infected U0126-treated HEp-2 cells and the controls were harvested at 3, 9 and 24 h p.i., and the samples were subjected to nuclear and cytoplasmic protein extraction. Equal amounts of extracts were electrophoretically separated, transferred to nitrocellulose sheets, and probed with antibodies recognizing a phosphorylated form of CDK (p-CDK-T14/Y15), phosphorylated form of p-cyclin E (p-cyclin E) and the late HSV-1 protein Us11. Anti-β actin and anti-Histone H3 were used as a housekeeping gene. Results demonstrated a decrease in the phosphorylated form of cyclin E in the infected cells treated with U0126, compared to the infected untreated cells in both cytoplasmic and nuclear fractions (Fig. 3a, lanes 8 and 12). In addition, in the cells infected with HSV-1, p-cyclin E was accumulated in both nuclear and cytoplasmic fractions (Fig. 3a, lanes 6 and 10). The U0126 treatment effectively reduced the levels of p-CDK (T14/Y15) (Fig. 3a, lanes 8 and 12). However, at 3, 9, and 24 h a clear decrease of p-CDK accumulation mainly in the nuclear fractions, was observed in the infected untreated cells (Fig. 3a lanes 2, 6, 10 and 12). In the cells treated with U0126, the decrease of phosphorylated form of cyclin E correlated with a decreased synthesis of the late viral protein Us11 in both cytoplasmic and nuclear fractions at 24 h (Fig. 3a, lane 6 vs lane 8 and lane 10 vs lane 12). To find out whether the activity mediated by U0126 on cyclin E and CDK2 correlated to a decrease in viral replication, the virus yield was determined. HEp-2 cells were pre-treated or untreated for 1 h at 37 °C with U0126 (5 µM), infected with HSV-1 at MOI 10 and collected at 24 h pi. Virus yield was determined by standard plaque using VERO cells monolayers, as described in Methods. The results showed a plain impairment of viral replication in cells treated with the inhibitor U0126 (*P ≤ 0.05) compared to the control (Fig. 3b) suggesting that the inhibition of MAPK activity negatively affects the ability of HSV-1 to replicate in our cellular model.

### ERK1 function is required in the control of cyclin E and CDK2 activation

**Figure 4:**
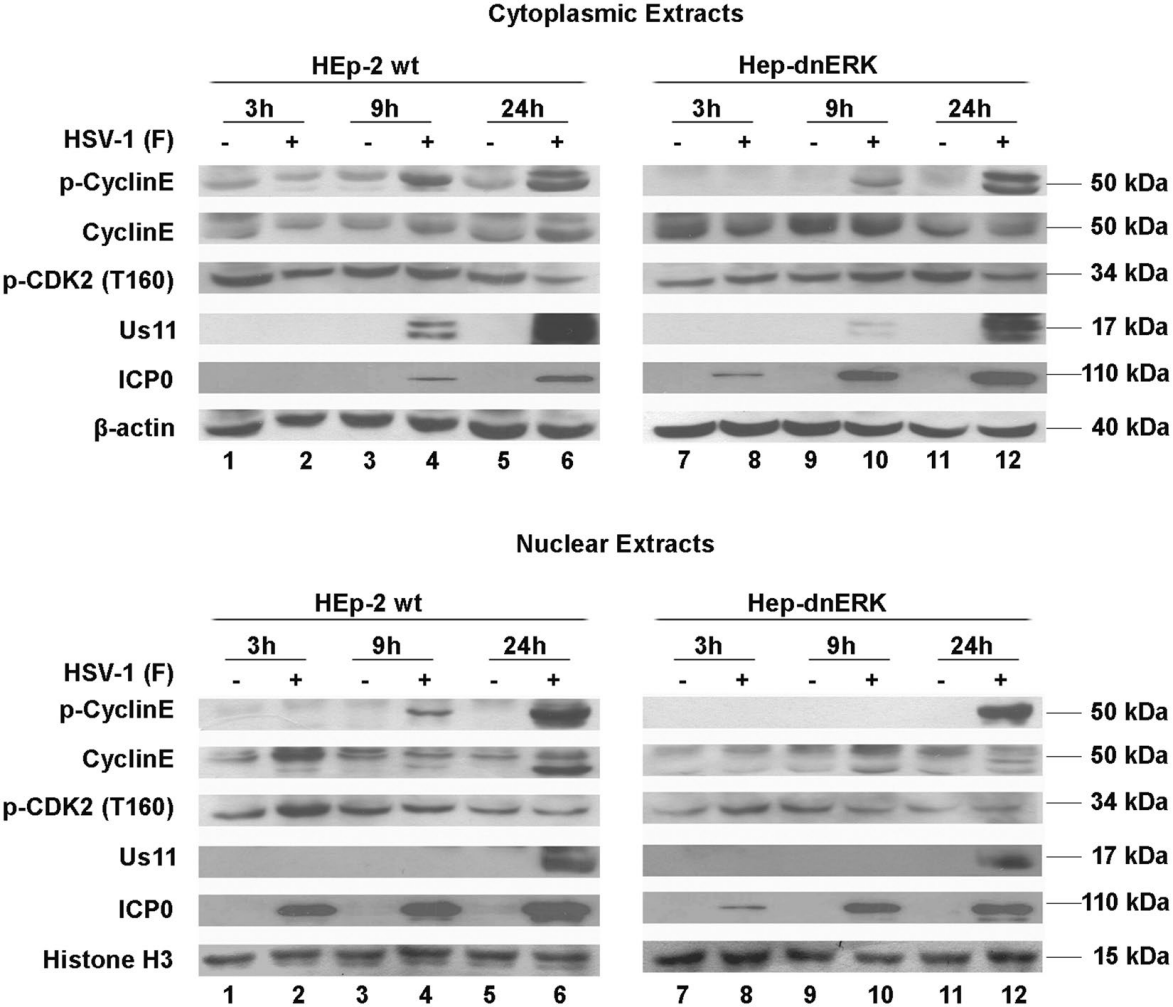
Cyclin E and CDK2 expression in dominant negative-ERK cells during HSV-1 replication. HEp-dnERK and HEp-2 cells were exposed to HSV-1 at MOI 10 and collected at 3, 9 and 24 h p.i. Cells were processed for cytoplasmic and nuclear proteins extraction as described in Methods and were analyzed by immuno-blot for the expression of total protein cyclin E, phospho-cyclin E, phospho-CDK2 (T160), Us11 and ICP0 proteins. β-actin and Histone H3 were used as cytoplasmic and nuclear loading controls, respectively. The filters derived from extracts obtained from both HEp-dnERK and HEp-2 cell lines were exposed simultaneously.

To better support the data obtained with an inhibitor of MEK pathways, dominant negative HEp-2 (HEp-dnERK) cell lines lacking in the normal function of ERK1 protein, were used. Experimental infections were performed, using HEp-2 and HEp-dnERK cells to directly correlate the regulation of cyclin E and CDK2 levels with the inhibition of ERK1 activity. HEp-2 and HEp-dnERK cell lines were infected with HSV-1 at MOI 10 and harvested at 3, 9 and 24 h p.i. Cytoplasmic and nuclear proteins were electrophoretically separated and probed with p-CDK2 (T160), p-cyclin-E and cyclin-E. The β-actin and the Histone H3 were used as housekeeping genes. The p-CDK2 (T160) antibody was chosen to detect the phosphorylated site T160 of CDK2, a target of ERK1/2 Kinases^19^. The results obtained demonstrated a decreasing accumulation of total and phosphorylated form of cyclin E in the HEp-dnERK infected cells compared with the HEp-2 wt cells, particularly in nuclear fractions (Fig. 4 lanes 10 and 12 vs lanes 4 and 6). Using an antibody against the phosphorylated site of CDK2 (T160) at 3 h p.i., a quantitative analysis revealed an accumulation of pCDK2 (T160) in the nuclear fraction of HEp-2 cells compared to the HEp-dnERK cells (Fig. 4 lane 2 vs lane 8). It is remarkable to note that this data correlates with the translocation of p-ERK1/2 from cytoplasm into the nucleus at 3 h post HSV-1 replication in HEp-2 cells (Fig. 2a lane 10). In addition, in HEp-dnERK cells, where ERK1 activity was impaired, the accumulation of immediate early (ICP0) and late (Us11) viral proteins was considerably reduced. Indeed at 3 h and 9 h after infection the nuclear accumulation of ICP0 was more abundant in HEp-2 cells than in HEp-dnERK cells. Interestingly, in the HEp-2 cells the accumulation of ICP0 protein was mainly localized in the nucleus (Fig. 4 lanes 2, 4 and 6). In contrast, in HEp-dnERK cells the ICP0 protein was uniformly localized in both nuclear and cytoplasmic fractions. These data imply that the ICP0 and ERK1/2 nuclear localization coincides temporally, underlining that the virus could probably requires ERK1 functions or that ICP0 requires ERK1 activity. The Us11 protein was more highly accumulated at early time point (9 h) in HEp-2 cells than in HEp-dnERK cells (24 h), mainly in the cytoplasmatic fractions (Fig. 4).

### Impaired virus growth in a cell line expressing a dominant negative form of ERK1

**Figure 5:**
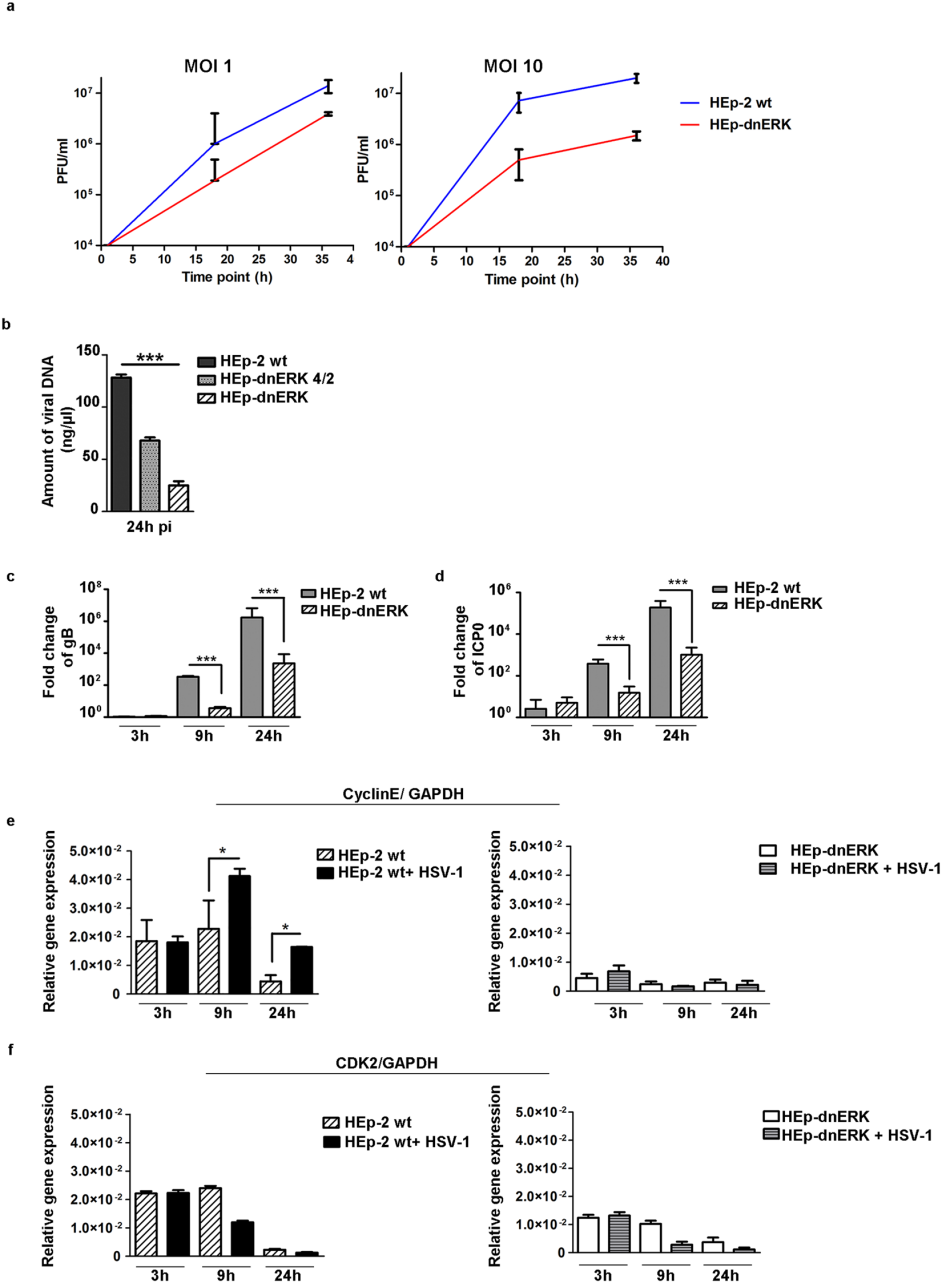
Effect of modulated ERK pathway on HSV-1 replication. (**a**) MOI-dependent growth curves were obtained by infecting HEp-2 and HEp-dnERK with HSV-1 at MOI 1 and 10. The sample were harvested at 1 h, 18 h and 36 h pi, the virus yields were measured by standard plaque assay on Vero cells. The procedure was described in Methods. (**b**) HEp-2, HEp-dnERK and HEp-dnERK 4/2 cell lines were infected with HSV-1 at MOI 10 and collected 24 h pi. The viral DNA was extracted 24 h post HSV-1 infection as described in the Methods. Viral DNA amounts were determined by absolute real-time PCR using a TaqMan probe. The result is expressed as concentration in ng of DNA/µl. (**c–f**) Quantitative Real-Time PCR of gB, ICP0, CyclinE and CDK2 transcripts in HEp-2 and HEp-dnERK cells were evaluated at 3 h, 9 h, 24 h pi. Calculations of the copies number were based on external standards and all transcripts were normalized against the GAPDH copies number. Statistical analyses were performed with one-way ANOVA analysis assay in triplicate and *P ≤ 0.05, **P < 0.01 ***P < 0.001 indicate significant change”.

As established above, the proteins necessary for cell cycle progression such as cyclin E and CDK2 during HSV-1 infection were negatively regulated in dominant negative ERK1 cells as well as in the U0126 treated cells. The incapability of virus to activate cyclin E and CDK2 in the absence of ERK and the reduction of viral protein expression allowed us to suppose a downstream effect on viral replication efficiency. To this end, we designed experiments to determine the capability of HSV-1 to replicate in the absence of a functional ERK1 protein. HEp-2 wt and HEp-dnERK were infected with HSV-1 at MOI 1 and 10 as described in Methods. The sample were harvested at 1 h, 18 h and 36 h pi and the virus yields were measured by standard plaque assay on Vero cells. The growth curves in Fig. 5a show a significant incapability of HSV-1 to replicate in HEp-dnERK if compared to the HEp-2 wt at all time considered in both low and high MOI (**P < 0.01 ***P < 0.001 respectively). Furthermore, in a parallel experiment, viral DNA quantification by absolute real-time PCR was evaluated in parental cell, in HEp-dnERK and in a clone identified as HEp-dnERK 4/2 which expresses low levels of dnERK1 protein. The three cell models were infected with the HSV-1 virus at MOI 10 and collected at 24 hours later. Absolute quantification by real time PCR was used to measure the copy numbers of viral DNA in the cell lines considered. These experiments were performed to identify the potential defects in viral DNA accumulation when ERK1 was affected with different degree. As shown in Fig. 5b, a significant decrease in viral DNA copy numbers was detected in HEp-dnERK cells infected by HSV-1 if compared to the HEp-2 parental cells. To note, Fig. 5b shows also that the decrease in viral DNA copy numbers was directly correlate with the inhibition of ERK functions. Indeed clone HEp-dnERK 4/2, which ﻿expresses low levels of dnERK1, displayed a limited decrease in viral DNA copy numbers if compared to the clone with higher levels of dnERK1 expression (***P < 0.001). Lastly the levels of viral and cellular transcripts such as ICP0, gB, cyclin E and CDK2, were analyzed by relative quantitation real-time PCR. The virus yields, measured by plaque assay, demonstrated that the viral growth in HEp-dnERK was impaired if compared to the parental cells (Fig. 5a). In addition, absolute quantification real time PCR was used to measure the number of copies of viral DNA in both cell lines. The experiments were performed to identify the potential defects in viral DNA accumulation when the ERK1 was affected. Total DNA was extracted at 24 h p.i., from HEp2 and HEp-dnERK cells infected with HSV-1 at MOI 10 and the number of HSV DNA copies in each sample was quantified as described in Methods. As shown in Fig. 5b, a significant decrease in viral DNA copy numbers was detected in HEp-dnERK cells infected by HSV-1 if compared to the amounts of HSV-1 DNA in the HEp-2 parental cells. Lastly, to determine whether the lower accumulation of DNA correlated with the decrease of the viral genes transcription, we analyzed gB and ICP0 viral transcripts accumulation either in parental cells or in those expressing dnERK upon infection with HSV-1. Interestingly, as shown in Fig. 5c-d, a significant decrease in the copy numbers of the ICP0 and gB viral transcripts was detected in cells expressing dnERK compared to the parental cells. Besides, we analyzed the expression of a cellular genes cyclin E and CDK2.

The Fig. 5e shows a different temporal expression of cyclin E transcripts in HEp-2 infected with HSV-1 compared to the mock infected cells. Indeed the levels of the cyclin E transcripts increased upon HSV-1 infection at 9 and 24 h p.i. (*P ≤ 0.05). It is interesting to note that in HEp-dnERK cells, the levels of the cyclin E transcripts remain unchanged upon viral infection and during all considered time. Since we had observed up regulation of cyclin E transcripts even at late time post infection (24 h) we analyzed also the transcripts levels of CDK2 cellular gene related to cyclin E function. The transcripts remain unchanged, upon viral infection, at 3 and 24 hrs and only at 9 h p.i. we noted a decrease of CDK2 transcripts levels. This suggest a specific control mediated by the virus on cyclin E transcripts.

### G1 phase is associated with the expression of the recombinant dnERK protein in infected cells

**Figure 6:**
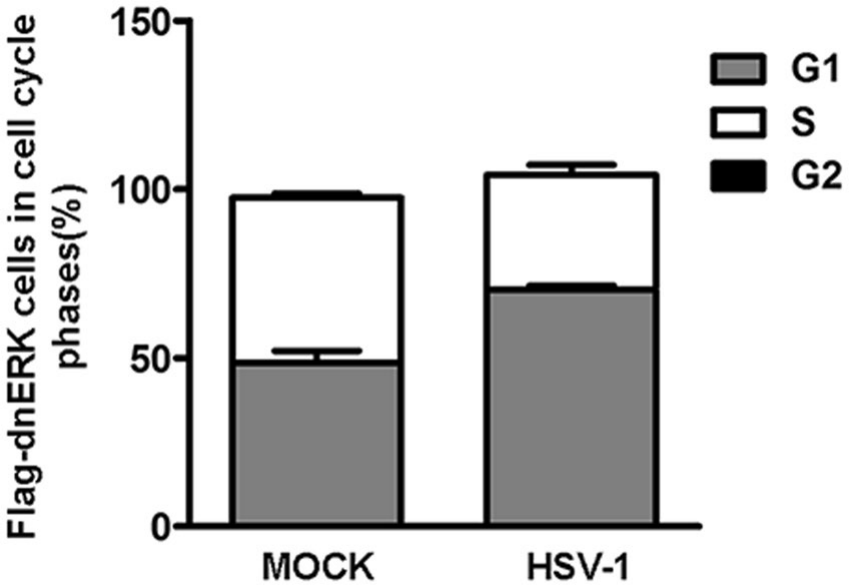
Correlation between FLAG-dnERK mutant/DNA-content in the infected cells. HEp-dnERK cells, containing an N-terminal FLAG tag in the ERK1 CDS, were mock infected or infected with HSV-1 at MOI 10 and collected at 24 h pi. The cell cycle phase of the FLAG-dnERK mutant cells with/without HSV-1 infection was determined by FACS analysis as described in Methods. Dual-parameter FLAG-dnERK mutant/DNA-content analysis were analyzed using Flowing Software 2.5.0. The percentages of cells distributed in S, G1, G2/M phases are shown in FLAG-dnERK mutant-positive cells. HEp-2 wild type cells not expressing FLAG-dnERK were used as a control to setting instrument. The data are shown as means of triplicate ± SD.

To directly link the cell cycle phase and the presence of mutated ERK1 protein at single cell levels during HSV infection, double labeling using an anti-Flag antibody recognizing the dnERK1 recombinant protein and propidium iodide staining was employed. In this condition, it was possible to identify the distribution of cell populations in each cell cycle phase (G1, S and G2/M phases) in all flagged-dnERK HEp-2 cells. The HEp-dnERK cells, mock infected or infected with HSV-1 at a MOI 10, were analyzed at 24 h p.i. by flow cytometry. The HEp-2 wild type cells, were used to measure the background fluorescence of Flag-negative cells. The results demonstrated that at 24 h p.i. most of the cells infected with HSV-1 lacking ERK function were largely blocked in G1 phases (70.2%) and not in S phase (29.8%). Beside the HEp-dnERK uninfected cells were equally distributed in G1 phases (48.5%) and S phases (49.0%) (Fig. 6). These results confirms that ERK1 activity is fundamental to control cell cycle upon HSV-1 infection in HEp-2 wild type cell lines.

## Discussion

Several studies have shown that replication of HSV and other large DNA viruses requires cellular proteins normally regulated in a cell cycle dependent manner, such as the cyclin-dependent kinases (cdks). In particular, data from different laboratories have reported the effects of HSV-1 infection on cellular proteins involved in cell cycle regulation. Indeed high concentrations of inhibitors of cdk2, cdc2, and cdk5 affect HSV-1 replication^2, 8, 36^. It was suggested that cdk-activated cellular and/or viral transcription factors might be required for optimal transcriptional activation of the viral genome^7^. All of these data show that HSV-1 is able to influence some key events occurring in G1/S phases.

However data from other groups demonstrated that Herpes simplex virus type 1 (HSV-1) infection disrupts cell cycle progression by two different ways and depending on the cell cycle phase of cellular model used. In quiescent cells, HSV-1 infection prevents G1 entry by inhibition of cyclin D/CDK4,6-specific and cyclin E/CDK2-specific phosphorylation of pRb. On the other hand, in dividing cell cultures, HSV-1 induces G1/S arrest by inhibition of preexisting cyclin E/CDK2 and cyclin A/CDK2 activities^26, 37^. In addition enrolment of mutant virus, in which ICP0 is the only IE protein expressed, resulted in p53-independent cell cycle arrest in G1/S and G2/M phase^9, 10^.

The above early discovers seems to be contradictory. However we demonstrated that HSV-1 infection significantly addresses (*P* < 0,001) the cell cycle progression through the S phase in asynchronized HEp-2 cells, and that the block of viral replication by PAA, alters this phenotype (Fig. 1). This kind of behavior is generally associated to DNA tumor viruses belongs to the Herpesviridae family, which have evolved mechanisms to force quiescent cells to enter into S phase creating an environment more advantageous to their replication. To date, this viral strategy was not associated to α-Herpesvirus such us HSV type 1. In our work, we also demonstrated that in the asynchronized HEp-2 cellular model, the molecular mechanisms involved in cell cycle progression through S phase were related to the recruitment of phosphorylated form of cyclin E and that HSV-1 infection leads to a decrease of the phosphorylated form of CDK protein.

Additionally, we highlight for the first time the fundamental association between HSV-1 and ERK1/2 recruitment as well as the ERK1/2 compartmentalization during active replication. Indeed, we demonstrated that within the first 24 h of viral replication in HEp-2 cells, HSV-1 leads to ERK1/2 proteins phosphorylation with a different cellular localization. The most crucial finding was that during the first phase of viral replication, HSV-1 prevents p-ERK1/2 nuclear translocation with resultant localization into the cytoplasm. In the late phase of infection, during the active transcription of viral late genes, the virus induces a full p-ERK1/2 translocation into the nucleus. It is remarkable to note the incapability of HSV-1 to control p-ERK1/2 accumulation in a spatio-temporal fashion in presence of PAA (Fig. 2b).

Accumulating evidence suggests that the spatial- temporal distribution of ERK1/2 can affect the qualitative and quantitative features of downstream signaling and cellular events^38^.

Interestingly, other studies have clearly associated high ERK activation with programmed cell death rather than with cell cycle progression^39^. Furthermore, results by others have demonstrated that ERK activation controls CDK2-cyclin E activity independently of its effects on regulation of cyclin D1 expression and CDK4-cyclin D1 activation^40, 41^. In our model, the prolonged ERK1/2 phosphorylation and subsequent nuclear translocation correlated downstream with the recruitment of cyclin E and the dephosphorylation of CDK2 protein in Thr-14 and Tyr-15 in the late phase of viral replication upon HSV infection. Indeed, in our conditions the cyclin E phosphorylation increased at 9 h after HSV-1 incubation, reaching maximal levels at 24 h (Fig. 3).

Several reports have demonstrated that the cyclin E enrollment can be sufficient to activate the CDK2-cyclin E complex in late G1, enabling cells to progress into the S phase and replicate cellular DNA^42, 43^. Based on this we established the contribution of ERK1/2 in cyclin E regulation and in G1/S cell cycle progression mediated by the HSV-1 virus. Indeed, by using either U0126 or dominant negative HEp-dnERK cell line, we found a clear influence on cyclin E expression, as expected. The inhibitor prevents HSV-induced cyclin E accumulation either into the cytoplasm or into the nucleus upon HSV infection (Fig. 4). The inhibition of ERK markedly reduced the levels of the viral gene expression and the viral yield. These data imply that ERK activity has an additional role in controlling cyclin E expression via its effect on HSV replication.

Our model is different from the model established in serum-deprived NHDFs, where the HSV-1 Us3 protein kinase inhibited ERK1/2 phosphorylation late during viral replication^29^. However, our results are in agreement with previous studies where all specific inhibitors of Ras/ERK pathways inhibit the viral replication through an unidentified mechanism^44–46^. In addition, we demonstrated that in HEp-dnERK cells ICP0 protein was mainly localized in the cytoplasmic fraction rather than in the nuclear fraction, if compared to the wild type cells (Fig. 5). It is well known that infection by HSV-1 is able to block cells at the mitotic stage of the cell cycle and this mitotic block is specifically due to ICP0 IE protein^10^. Besides, during the course of infection, ICP0 localizes into the nucleus and disrupts ND10 bodies, followed by inhibition of host chromatin silencing, mechanisms that in turn block the transition from immediate-early to early gene expression^47^. Our results suggests the potential ERK1 role on the ICP0 function which affect downstream both failure of the virus to control cell cycles and failure to control the second wave of viral transcripts that culminate in a defective viral replication.

Lastly, the use of an anti-Flag antibody to recognize dnERK harboring cells and propidium iodide staining, allowed us to identify the changes in the cell cycle phases of the infected cells. Indeed at 24 h post infection, most of the cells infected with HSV-1, lacking ERK1 function, were mainly blocked in G1 phase as compared to the uninfected cells (Fig. 6). In conclusion the present data add a new perspective on the capability of HSV-1 to regulate the cell cycle G1/S transition and the state of cyclin E/CDK2 in HEp-2 cell, indicating the mechanistic interactions between cyclin E expression and an intermediary role of ERK during HSV-1 replication. Such regulation seems to be an essential prerequisite for efficient replication and is essential for virus yield.

## Methods

### Cell culture

VERO cell lines (American Type Culture Collection) were propagated in minimal essential medium (MEM), supplemented with 6% fetal bovine serum (FBS) (Lonza, Belgium) and mixture 100 U/ml penicillin and 100 mg/ml streptomycin. HEp-2 cells (human larynx epidermoid carcinoma cell line) and transfectans derived from HEp-2 parental cell lines, designed HEp-dnERK (where dn means dominant negative), were grown in Dulbecco’s modified Eagle’s medium (Gibco/Invitrogen Corporation, Grand Island, NY) supplemented with 10% of fetal bovine serum, 100 U/ml penicillin and 100 mg/ml streptomycin. Geneticin (400 µg/ml) was used to maintain HEp-dnERK under selection. All cell lines were incubated at 37 °C under 5% CO_2_.

### Viral infection

HSV-1 (F) is the prototype HSV-1 strain kindly provided by Professor Bernard Roizman. Virus stocks were propagated and then titered in Vero cells.The experimental infections were carried out by exposure of HEp-2 and HEp-dnERK cells to the HSV-1 virus at the multiplicity of infection (MOI) of 10. After the infection, the cells were placed at 37 °C with gentle shaking. After 1 h, the supernatant was replaced with fresh culture medium, and then the infected cultures and related controls were incubated at 37 °C, under 5% CO_2_, and collected at the established times of the experimental design.

### Antibodies

Polyclonal antibodies against the housekeeping gene β-actin and GAPDH were purchased from Abcam and Santa Cruz Biotechnology, respectively. Histone H3 and phospho-ERK1/2 (Thr 202/Tyr 204) (20G11) were purchased from Cell Signaling Technology (Beverly, MA). Antibodies to the phosphorylated forms of cyclin E (sc-12917-R), CDK (sc-28435-R), CDK-2 (sc-101656), total form of cyclin E (sc-247), and viral protein ICP0 (sc-56985) were purchased from Santa Cruz Biotechnology (Santa Cruz, CA), Us11 antibody was kindly provided by Professor Bernard Roizman. ERK1/2 (137F5) was purchased from Cell Signaling Technology. Monoclonal antibody to FLAG FITC-conjugate was purchased from SIGMA. Secondary antibodies anti-rabbit and anti-mouse IgG conjugated to peroxidase were also from Santa Cruz Biotechnology. Protein bands were visualized using SuperSignal West Pico as a chemiluminescent substrate (Thermo Scientific, Rockford, IL).

### Inhibitors

U0126, specific inhibitor of the MEK1/2^48^ was obtained from Cell Signaling Technology (Beverly, MA) and used at the final concentration of 5 µM. U0126 has been reconstituted in DMSO; the same percentage of DMSO, diluted in RPMI, was used in the untreated cells. HEp-2 cells, were pre-treated for 1 h at 37 °C with U0126 (5 µM), and infected with 10 PFU of HSV-1 in presence of the inhibitor. Phosphonoacetic acid (PAA) from Sigma-Aldrich, was used as an inhibitor of DNA synthesis in HSV-infected cells and was dissolved in the medium and used at 300 µg/ml during and after HSV-1 adsorption. At different time points post infection, defined in experimental designs, the cells were harvested and analyzed.

### Standard Plaque Assay on VERO cells

Confluent monolayers of VERO cells were prepared in 12 multiwell plates. The infected samples were frozen and thawed three times and diluted. Hundred µl of each dilution of the suspension were used to infect the monolayers. The multiwell plates were incubated for 1 h at 37 °C. Then viral inoculum was removed and 1 ml of culture medium containing 0.8% methylcellulose was added. After 72 h the plaques were visualized and counted at the microscope after staining with a crystal violet solution.

### Construction of dominant negative ERK HEp-2 cells

The plasmid, harboring N-terminal FLAG-ERK1 CDS, containing a mutations on K^72^-R, T^202^-A and Y^204^-F designed pcDNA-dnERK was previously described^49^. Briefly semi-confluent cultures of HEp-2 cells were transfected according to the following experimental procedure: 2.5 × 10^5^ cells/well were implanted in 6-well plates with D-MEM 10% FBS. After 24 h the medium was replaced with D-MEM medium without serum and antibiotics, pcDNA-dnERK plasmid, described above, was incubated with Plus Reagent (Invitrogen) and 96 µl of OPTIMEM (Invitrogene) at room temperature (RT) for 20 min. Lipofectamine (Invitrogen) was added to 96 µl of OPTIMEM. The two solutions were then mixed and incubated at RT for 30 min. The DNA-lipofectamine mixture obtained, was slowly added to the cell cultures and incubated for 4 h at 37 °C. Then, 500 µl of OPTIMEM with 10% of FBS were added. After 72 h, cells were trypsinized and cultured in presence of the selective agent geneticin (G418) at final concentration of 400 μg/ml. The resistant cells designed as HEp-dnERK were amplified and subsequently assayed for the expression of recombinant dnERK-N-terminal FLAG by immunofluorescence assay. HEp-dnERK and the HEp-dnERK 4/2 clones,expressing respectively higher and lower levels of ERK1, were used.

### Protein extractions and Immunoblot

Immunoblot analysis was performed lysing cell lines in SDS Sample Buffer 1x (62.5 mM Tris-HCl pH 6.8; DTT 1 M; 10% glycerol; 2% SDS; 0.01% Bromophenol Blue) to total proteins extraction. To separate cytoplasmic from nuclear extracts the samples were collected and resuspended in 150 μl of hypotonic buffer A [10 mM HEPES, pH 7.9, 1.5 mM MgCl_2_, 10 mM KCl, 0.5 mM dithiothreitol (DTT)], 0.2 mM phenyl methyl sulfonyl fluoride (PMSF), 1X Protease inhibitor (Roche Applied Science). The cell suspensions were then incubated on ice for 15 min. and were lysed passing through a 25-gauge needle for 15 times. Cytoplasm fractions were collected by centrifugation at 12000 rpm for 5 min. at 4 °C. Cell pellets were then washed, centrifuged and resuspended in 15 μl of Buffer B [20 mM HEPES, pH 7.9, 25% Glycerol, 0.42 M NaCl, 1.5 mM MgCl_2_, 0.2 mM EDTA, 0.5 mM DTT, 1 X Protease inhibitor]. Nuclear fractions were collected by centrifugation at 12000 rpm for 5 min. at 4 °C. Equal amounts of protein extracts were used for western blot analysis to evaluate the accumulation of both viral and cell cycle regulatory proteins.

### RNA extraction and Reverse Transcription

Total RNA was extracted using TRIzol® (Life Technologies) according to the manufacturer’s instructions. Total RNA was DNAse-treated before cDNA transcription as follows: 8 µl of total RNA was incubated with 1X DNAse buffer, 1 unit DNAse and 0.8 units RiboLock RNase inhibitor (Fermentas, part of Thermo Fischer Scientific, Waltham, MA, USA) at 37 °C for 30 min., 1 µl EDTA (stop-solution) was added and samples were incubated for 10 min. at 65 °C. Total RNA was reverse transcribed using RevertAid H Minus M-MLV Reverse transcriptase (Fermentas). The RT reaction was carried out in PerkinElmer PCR machine (Waltham, MA, USA) under the following conditions; at 42 °C for 60 min. followed by 90 °C for 5 min. The cDNA was stored at – 70 °C and used for quantitative real-time RT-PCR.

### DNA extraction

HEp-2 and HEp-dnERK were infected with 10 PFU of HSV-1 (F) and twenty-four hours after infection were resuspended in 1 ml of TRIzol RNA/DNA/protein isolation reagent and used for DNA extraction, according to the manufacturer’s instructions. The DNA was precipitated from the interphase and organic phase as described in ref. 30.

### Real-time PCR

Quantitative real-time RT-PCR was performed with Rotor-Gene 6000 real-time instrument (Corbett Research, Mortlake, Victoria, Australia) using Maxima SYBR Green (Fermentas) as described by^50^^.^ Shortly, the PCR reaction was carried out under the following conditions: initial denaturation at 95 °C for 15 sec., 45 cycles at 95 °C for 15 sec./55° or 60 °C for 30 sec./72 °C for 45 sec., followed by generation of melting curve from 72 °C to 95 °C. The annealing temperature was chosen based on the primer sequences, with 55 °C for GAPDH and 60 °C for ICP0, gB and cyclin E. The cDNA copy numbers were normalized to GAPDH^51, 52^. The analytic primers for RT-PCR are following: gB Fw-5′tagctggtgtgttcggtgtg3′ and Rev-5′ggacgacggtaaactgcatc3′; ICP0 Fw-5′tctgcatcccgtgcatgaaa3′ and Rev-5′cacgcccactatcaggtaca3′; Cycline E Fw-5′gaagaggaaggcaaacgtga3′ and Rev-5′tgcacgttgagtttgggtaa3′. CDK2 Fw-5′cattcctcttcccctcatca3′ and Rev-5′atggccccctctgtgttaat3′. Each quantitative Real-time PCR experiments include a minus-reverse transcriptase control.

Absolute quantification Real-time PCR using specific TaqMan probe was performed to detect viral DNA. Viral load was derived from the threshold cycle (*CT*) using the standard curve generated in parallel and the result is expressed as concentration in ng of DNA/µl. The procedures were published previously^30^.

### FACS Analysis

The cells were harvested, washed in PBS and transferred into polystyrene tubes. The cells were fixed in cold 70% ethanol for 2 h at 4 °C. Then cells were resuspended in Propidium iodide (PI) staining solution (0.1% [v/v] Triton-X100, 10 µg/ml PI, 100 µg/ml DNase free-RNase A in 1x phosphate saline buffer [PBS]) and incubated in at 37 °C for 10 min. in the dark. The samples were acquired and analyzed using flow cytometer (BD FACScalibur). The results were analyzed using the software for the analysis of cell cycle (ModFit LT version 3.0).

To detect simultaneously HEp-dnERK FLAG-positive cells and the cell cycle phases of the cells, the HEp-dnERK and parental cell lines were infected with HSV-1 and double labeled.

The anti FLAG-primary Ab was diluted (1:100) and incubated with cells for 60 min. at RT. Samples were then washed and incubated at RT for 30-60 min. with fluoresceinated secondary Ab (1:200 dilution FITC conjugate). The samples were acquired and analyzed using flow cytometer (BD FACScalibur). To detect double labelled cells Flowing Software version 2.5.0 was used.

### Statistical Analysis

Each experiment was repeated for at least three times. All Western Blot band intensities were normalized respect to the control level using T.I.N.A. software. The results are reported as means ± standard deviation and statistical calculations and graphical representation were done by One-way analysis of variance (ANOVA) using Prism software (GraphPad): *P ≤ 0.05, **P < 0.01 ***P < 0.001.

## Electronic supplementary material

Supplementary Information
