# Supplementary material for: The ERK-1 function is required for HSV-1-mediated G1/S progression in HEP-2 cells and contributes to virus growth
Source: Sci Rep. 2017 Aug 23;7:9176. doi: 10.1038/s41598-017-09529-y (PMC5569015; doi:10.1038/s41598-017-09529-y)
Supplement: Supplementary file 1 — Supplementary Information [file 41598_2017_9529_MOESM1_ESM.pdf]

The ERK-1 function is required for HSV-1-mediated G1/S progression in HEP-2 cells and contributes to virus growth

Ivana Colao[1], Rosamaria Pennisi[1], Assunta Venuti[3], Michaela Nygårdas[2], Outi Heikkilä[2], Veijo Hukkanen[2], Maria Teresa Sciortino[1#]

1) Department of Biological and Environmental Sciences, University of Messina, Viale F. Stagno d'Alcontres 31, 98166 Messina, Italy;
2) Department of Virology, University of Turku, Turku, Finland
3) Division of Immunology, Transplantation and Infectious Diseases, San Raffaele Scientific Institute, Milan, Italy

corresponding author. Maria Teresa Sciortino
email: mtsciortino@unime.it
phone number: +39 090 6765217

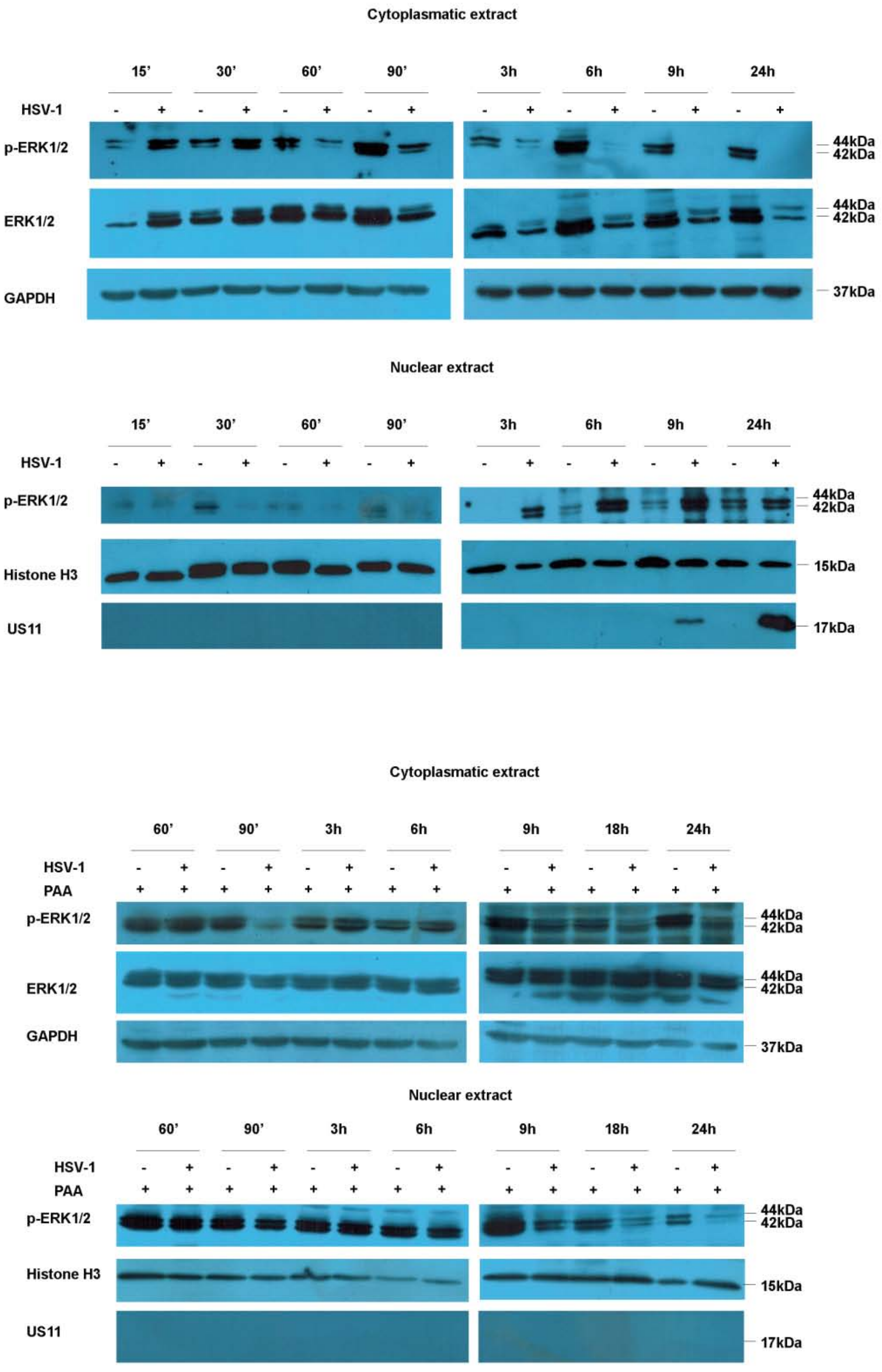

FIGURE 2

Figure 2: Expression of phospho-ERK1/2 proteins in HEp-2 cells infected with HSV-1 and PAA treated. (a) HEp-2 cells were infected or mock infected with HSV-1 at MOI 10 and collected at different times p.i. (15', 30', 60', 90', 3, 6, 9 and 24 hrs). Equal amount of cytoplasmic and nuclear proteins were separated by polyacrylamide gel electrophoresis and probed with phospho-ERK1/2 (p42/44), ERK1/2 and US11 antibody. GAPDH and Histone 3 proteins were used as housekeeping for the cytoplasmic and nuclear fraction, respectively. (b) Cells were infected or mock infected and

treated with 300 μg/ml phosphonoacetic acid (PAA) and collected at 60', 90', 3, 6, 9 18 and 24 hrs. PAA was added at the time of adsorption and maintained throughout the course of infection and processed as described in panel b. Band density was determined with the T.I.N.A. program, and was expressed as fold change over the appropriate housekeeping genes.

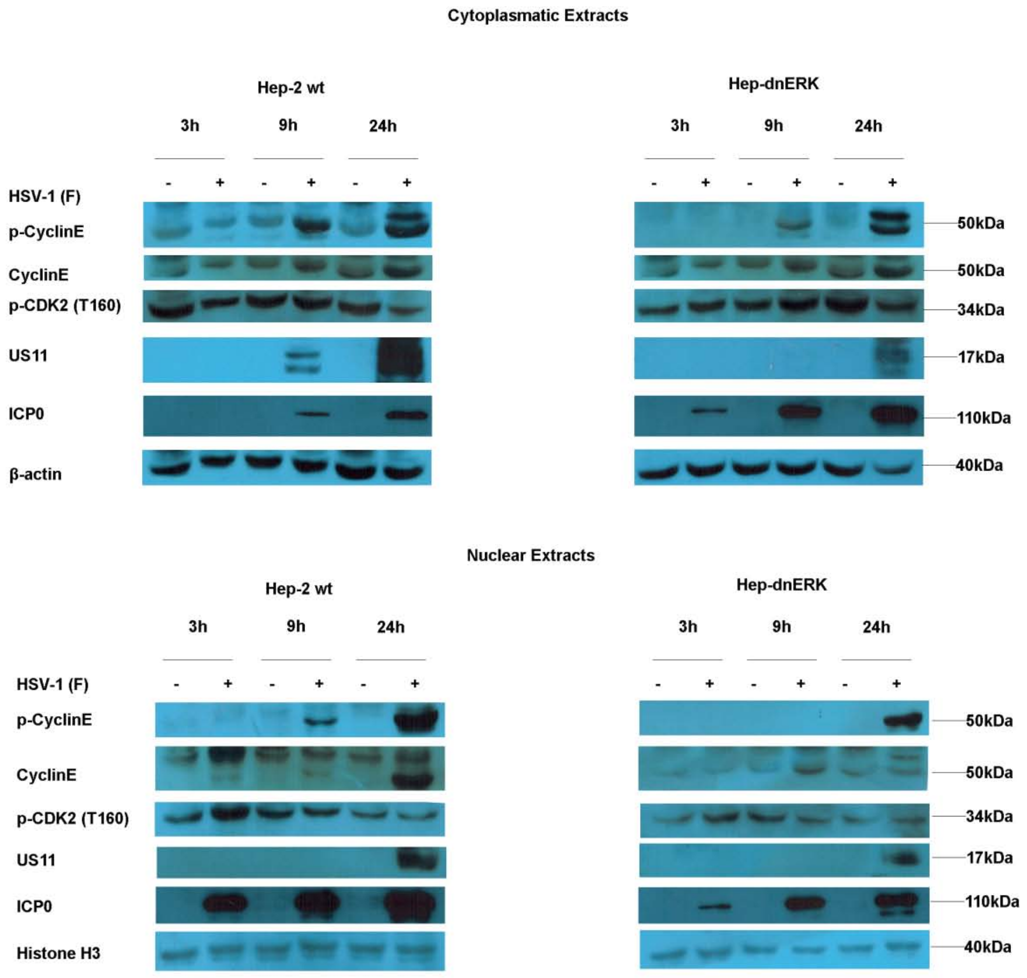

FIGURE 4

Figure 4: Cyclin E and CDK2 expression in dominant negative-ERK cells during HSV-1 replication. HEp-dnERK and HEp-2 cells were exposed to HSV-1 at MOI 10 and collected at 3, 9 and 24 h p.i. Cells were processed for cytoplasmic and nuclear proteins extraction as described in Methods and were analyzed by immuno-blot for the expression of total protein cyclin E, phospho-

cyclin E, phospho-CDK2 (T160), Us11 and ICP0 proteins. β-actin and Histone H3 were used as cytoplasmic and nuclear loading controls, respectively. The filters derived from extracts obtained from both HEp-dnERK and HEp-2 cell lines were exposed simultaneously.
